# Medical Education

**Published:** 1897-05-29

**Authors:** 


### MEDICAL EDUCATION.

An Earnest Inquirer writes asking what steps it is necessary to take to enter the medical profession.

⁂ Full information can be obtained on the subject by application to the Dean of any medical school, or, so far as the preliminary examination is concerned, by applying to the Registrar of the General Medical Council, 299, Oxford Street London, W.—Ed. *T. H.*

